# PERK/Sestrin2 Signaling Pathway Mediated Autophagy Regulates Human Cardiomyocytes Apoptosis Induced by Traffic-Related PM_2.5_ and Diverse Constituents

**DOI:** 10.3390/ijms26083784

**Published:** 2025-04-17

**Authors:** Jiayu Tian, Zeyu Niu, Huan Yang, Caihong Wang, Linlin Guan, Lifang Zhao, Dongxing Shi, Zhihong Zhang

**Affiliations:** 1 Department of Environmental Health, School of Public Health, Shanxi Medical University, 56 Xinjian South Road, Taiyuan 030001, China; jiayutian17@163.com (J.T.); 15534319188@163.com (Z.N.); guanlinlin@sxmu.edu.cn (L.G.);; 2 Yellow River Basin Ecological Public Health Security Center, Shanxi Medical University, 56 Xinjian South Road, Taiyuan 030001, China; 3 MOE Key Laboratory of Coal Environmental Pathogenicity and Prevention, Shanxi Medical University, 56 Xinjian South Road, Taiyuan 030001, China

**Keywords:** traffic-related PM_2.5_, cardiotoxicity, PERK/Sestrin2, autophagy, apoptosis

## Abstract

Although the strong causal association between PM_2.5_ and cardiovascular disease has been extensively studied, the latent molecular mechanisms have not been entirely explained. The objective of this research was to assess the cardiotoxicity of Traffic-related PM_2.5_ (TRPM_2.5_), water-soluble components (WSC), and water-insoluble components (WIC) in human cardiomyocytes (AC16) and to investigate the underlying molecular mechanisms. Endoplasmic reticulum stress (ERS), autophagy, and apoptosis were activated 24 h after exposure to total-TRPM_2.5_, WSC, or WIC. WIC was predominantly related to cardiotoxicity compared to WSC. Sestrin2 is an upstream molecule in several signaling pathways, including those involved in autophagy and apoptosis. In this study, we found that the knockdown of Protein Kinase RNA-like Endoplasmic Reticulum Kinase (PERK) suppressed the expression of PERK, Sestrin2, Caspase-12, Caspase-3, LC3, and p62 in TRPM_2.5_-treated AC16 cells. These results indicate that ERS participates in the activation of autophagy and apoptosis through the PERK/Sestrin2 pathway. We found that inhibiting autophagy with 3-methyladenine (3-MA) decreased the expression of autophagy-related factors and aggravated apoptosis. These observations suggest that protective autophagy was initiated. Finally, our findings provide valuable insights into the molecular mechanism by which ERS might regulate autophagy through the PERK/Sestrin2 signaling pathway, and protective autophagy may be activated to relieve TRPM_2.5_ and component-mediated apoptosis in AC16 cells.

## 1. Introduction

According to the State of Global Air 2024, air pollution caused approximately 8.1 million deaths worldwide in 2021 [1]. PM_2.5_ is the key air quality metric in the World Air Quality Report [2]. There are multiple sources of PM_2.5_, each of which may produce different chemical compositions and physical properties. Traffic-related PM_2.5_ (TRPM_2.5_), which mainly results from exhaust emissions, brake and tire wear, and dust resuspension, is an urban air contaminant that receives special focus in densely populated areas [3]. Our previous research found that 16 types of polycyclic aromatic hydrocarbons (PAHs) in the collected TRPM_2.5_ accounted for 2.74% of the total mass of TRPM_2.5_, and 5 soluble anions accounted for 20.25% of the total mass of TRPM_2.5_ [4]. An epidemiological study showed that long-term exposure of local residents to black carbon emitted from traffic exhaust was related to the incidence rate of stroke [5]. Therefore, it is necessary to investigate the potential toxicity of TRPM_2.5_ and its components to human health.

The Global Burden of Disease 2021 showed that high systolic blood pressure was the largest risk factor for the global disease burden in 2021. Simultaneously, the principal cause of mortality globally in 2021 is ischemic heart disease [6]. Extensive epidemiological documentation indicates that contact with PM_2.5_ is directly related to cardiovascular disorders, among which are acute myocardial infarction and ischemic heart disease [7], atherosclerotic cardiovascular disease [8], and coronary artery calcification [9]. Taken together, PM_2.5_ linked cardiovascular diseases (CVD) aggravate the disease burden worldwide. Moreover, given that the underlying pathological process for the relationship between various components of PM_2.5_ and heart diseases has not been well studied, there is an immediate requirement to explore the mechanisms through which PM_2.5_ has an effect on heart disease more comprehensively.

Accumulating evidence indicate that diverse forms of cell death, such as apoptosis, autophagy, pyroptosis, and ferroptosis, can be triggered by PM_2.5_ [10,11]. According to reports, varying degrees of organelle damage can result in cell death, such as in the endoplasmic reticulum (ER), lysosomes, and mitochondria. [12,13,14]. Research has shown that endoplasmic reticulum stress (ERS) is pivotal in the development of numerous CVDs [15,16]. Under ERS conditions, unfolded protein responses can maintain cellular homeostasis through different pathways, including the Protein Kinase RNA-like Endoplasmic Reticulum Kinase (PERK) signaling pathway. During ERS, glucose regulated protein 78 (GRP78) dissociates from PERK, activating it to phosphorylate eukaryotic initiation factor 2 α (eIF2α), which globally reduces protein synthesis while selectively enhancing activating transcription factor 4 (ATF4) translation. ATF4 then translocates to the nucleus and upregulates C/EBP homologous protein (CHOP), a transcription factor that can trigger cell death under persistent ERS after initially helping cells adapt by reducing protein synthesis and activating stress response genes. It is reported that autophagy and apoptosis are induced by PM_2.5_ as a consequence of ERS [17,18]. Nonetheless, the mechanisms by which PM_2.5_ induces apoptosis and autophagy through ERS remain unclear.

Sestrin2, a novel stress-induced protein that is a conserved antioxidant and metabolic regulatory factor, can be induced by various injuries, including oxidative stress, ERS, DNA damage, hypoxia, and inflammation [19]. Sestrin2 levels are regulated by multiple transcription factors, such as nuclear factor erythroid2-related factor 2 (Nrf2), ATF4, and ATF6 [19]. In recent years, research has shown that Sestrin2 participates in the occurrence and development of cardiovascular events [20,21]. Accumulating evidence suggests that Sestrin2 activates autophagy by activating the AMP-activated protein kinase (AMPK) and mTOR signaling pathways, indicating that Sestrin2 is a positive regulatory factor of autophagy [22,23]. Accordingly, in the current research, we aim to investigate whether the PERK signaling pathway plays a pivotal role in the positive regulation of autophagy carried out by Sestrin2.

Interestingly, autophagy, an adaptive process that promotes the turnover of toxic molecules and organelles, plays a notable dual role in the development of diverse diseases and the maintenance of physiological homeostasis. Some previous evidence suggests that autophagy activation exacerbates the damage caused by PM_2.5_ [24,25], while there is also evidence suggesting that autophagy plays a role in protecting cells from the cytotoxicity caused by PM_2.5_ [26,27].

Given that some studies on PM_2.5_ have neglected its sources and components, and considering the insufficient research on the mechanism of PM_2.5_-induced autophagy, the purpose of our study was to explore whether TRPM_2.5_ and its components activate autophagy through the PERK/Sestrin2 pathway and to determine the role of autophagy in this process. The AC16 cell line is highly similar to natural human cardiomyocytes in terms of physiological characteristics. It can be extrapolated to the human body more effectively than cardiomyocytes, such as H9c2 and HL-1. Moreover, it has a stable phenotype that enables the acquisition of reproducible results. Therefore, the AC16 cell line was selected as a cell model, and water-soluble components (WSC) and water-insoluble components (WIC) of TRPM_2.5_ were extracted to probe into the effects of TRPM_2.5_ and its components on the cardiomyocyte and the latent mechanism. This exploration will provide persuasive evidence to clarify the possible toxicological mechanisms of TRPM_2.5_, WSC, and WIC in cardiotoxicity.

## 2. Results

### 2.1. TRPM_2.5_, WSC, and WIC Descended the Cell Viability Rates of AC16

**Figure 1:**
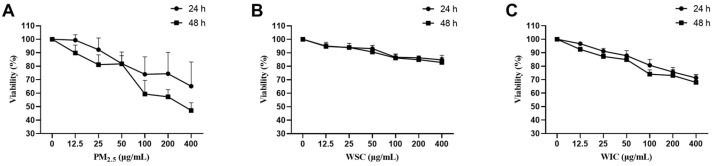
The viability of AC16 cells decreased after exposure to traffic-related PM_2.5_ (TRPM_2.5_), water-soluble components (WSC), and water-insoluble components (WIC) for 24 and 48 h. (**A**) Viability of AC16 cells stimulated by various concentrations of TRPM_2.5_ for 24 and 48 h. (**B**) Viability of AC16 cells stimulated by various concentrations of WSC. (**C**) Viability of AC16 cells stimulated by various concentrations of WIC. Data are shown as mean ± SD (*n* = 4).

The viability of AC16 cells was determined using the CCK-8 assay after treatment with gradually increasing doses (12.5, 25, 50, 100, 200, and 400 μg/mL) of TRPM_2.5_, WSC, and WIC for 24 and 48 h. The results implied that TRPM_2.5_, WSC, and WIC significantly inhibited cell viability (Figure 1), which imposed cytotoxicity on AC16 cells in a time- and concentration-dependent manner. We observed that TRPM_2.5_ particulate matter had the greatest impact on cell activity, with WIC being more severe than WSC exposure. Based on the experimental results and literature research [28], 50 and 100 μg/mL of TRPM_2.5_, WSC, and WIC were selected as administration concentrations for 24 h for the following assays.

### 2.2. TRPM_2.5_ and Components Induced AC16 Cells Apoptosis Through ERS

**Figure 2:**
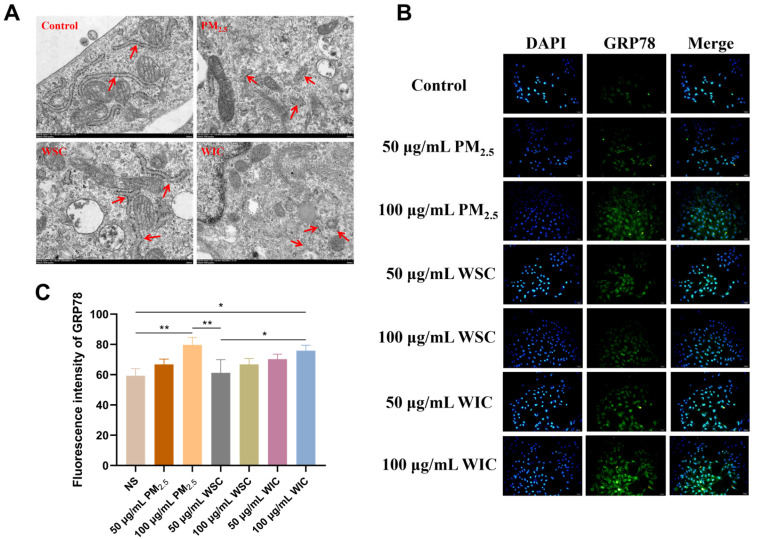
TRPM_2.5_ and its components expedited endoplasmic reticulum stress (ERS) in the AC16 cells. (**A**) Typical images of endoplasmic reticulum in AC16 cells by TEM (×20 k magnification). Red arrow: Changes in the endoplasmic reticulum structure. (**B**) IF staining of the level of GRP78 (200×). (C) GRP78 fluorescence intensity analysis. Data are shown as mean ± standard deviation (*n* = 3). * *p* < 0.05. ** *p* < 0.01.

TEM images of AC16 cells treated with 100 μg/mL TRPM_2.5_, WSC, and WIC showed some intracellular ultrastructure changes (Figure 2A). Significant ER swelling and fragmentation were observed in all cells of the PM_2.5_-treated group. Compared with the WSC group, the swelling and fragmentation of the ER were more pronounced in the WIC group.

The protein levels of GRP78 were detected using an IF experiment. After AC16 cells were exposed to TRPM_2.5_, WSC, and WIC for 24 h, the GRP78 levels in the 50 and 100 μg/mL TRPM_2.5_ groups were upregulated in a dose-dependent manner, as shown in Figure 2B,C. GRP78 levels in the WIC exposure group increased significantly (*p* < 0.01) compared with those in the control group; however, there was no significant difference between the WSC exposure and control groups. Furthermore, the expression was obviously higher after WIC exposure than after WSC exposure (*p* < 0.05). These results indicate that TRPM_2.5_ and WIC trigger ERS in AC16 cells.

**Figure 3:**
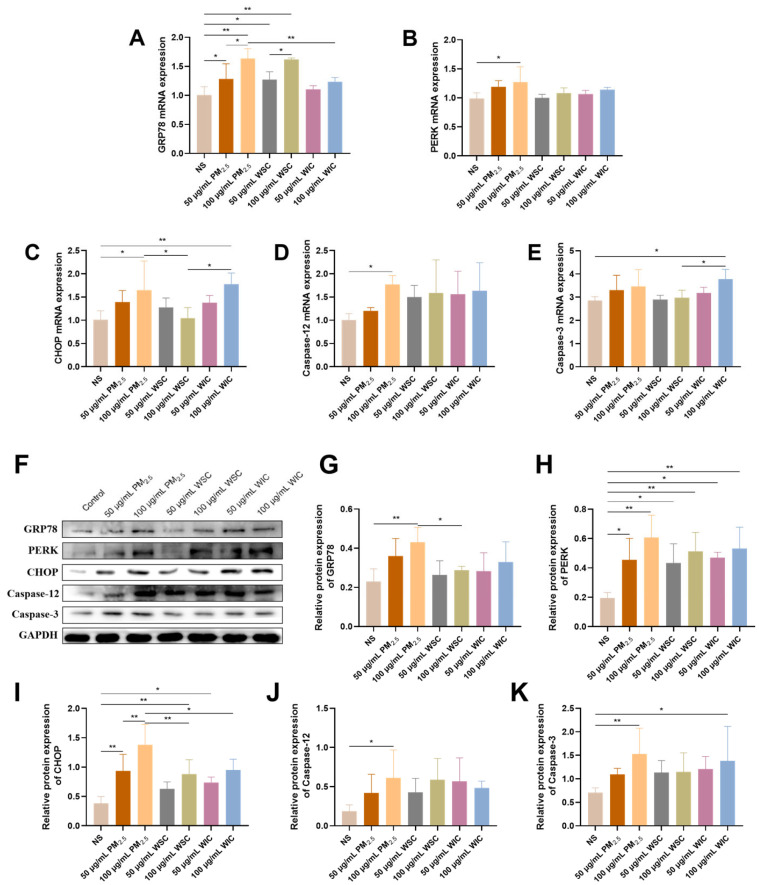
TRPM_2.5_ and its components induced apoptosis through ERS in the AC16 cells. (**A**–**E**) mRNA levels of GRP78, PERK, CHOP, Caspase-12, and Caspase-3 in AC16 cells. (**F**) Representative images of the relative protein expression of the above indicators. (**G**–**K**) Protein ray analysis of the above indicators. Data are shown as mean ± standard deviation (*n* = 3). * *p* < 0.05. ** *p* < 0.01.

To evaluate the effect of ERS on apoptosis levels stimulated by TRPM_2.5_, the gene and protein levels of GRP78, PERK, CHOP, Caspase-12, and Caspase-3 were examined. The qRT-PCR results indicated that TRPM_2.5_ was capable of upregulating gene expression related to ERS. Compared to the WSC group, WIC resulted in more rapid growth of *CHOP* and *Caspase-3* levels (Figure 3A−E). Corresponding to the RNA transcript, protein levels across all TRPM_2.5_ groups displayed substantial disparities (Figure 3F−K). The differences in PERK, CHOP, and Caspase-3 levels following TRPM_2.5_ component exposure were conspicuous, with WIC having a greater influence on CHOP and Caspase-3 protein expression levels compared to WSC. Our exploration revealed that the ERS signaling pathway was activated, leading to the occurrence of cell apoptosis after stimulation of TRPM_2.5_, WSC, and WIC in AC16 cells.

### 2.3. TRPM_2.5_ and Components Triggered AC16 Cells Autophagy

**Figure 4:**
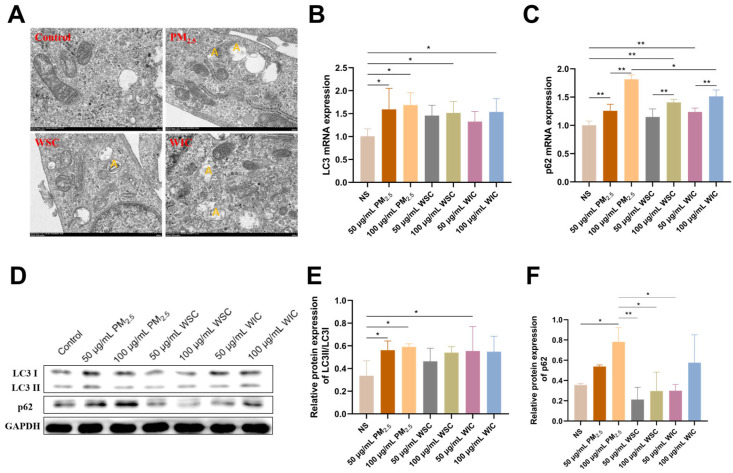
TRPM_2.5_ and its components activated autophagy in the AC16 cells. (**A**) Typical images of autophagosomes in AC16 cells observed using TEM (×20 k magnification). A: number of autophagosomes. (**B**,**C**) mRNA levels of LC3 and p62 in AC16 cells. (**D**) Representative images of the relative protein expression of the above indicators. (**E**,**F**) Protein gray analysis of the above indicators. Data are shown as mean ± standard deviation (*n* = 3). * *p* < 0.05. ** *p* < 0.01.

Autophagy is a prominent form of cell death; therefore, we further investigated the effects of TRPM_2.5_, WSC, and WIC on autophagy in AC16 cells. Cell ultrastructural damage was measured. TEM observation revealed that cells treated with TRPM_2.5_ and its components significantly exhibited excessive formation of autophagosomes compared to the control group, especially in the TRPM_2.5_ whole particle group, where a large number of autophagosomes were observed (Figure 4A).

Due to the clues provided by TEM, we next determined the levels of LC3 and p62, which are critical markers of autophagy, to validate whether TRPM_2.5_ and its components have the capacity to facilitate autophagy in AC16 cells. As shown in Figure 4B,C, qRT-PCR revealed that the levels of *LC3* and *p62* increased substantially after treatment with TRPM_2.5_, WSC, and WIC at 24 h in a dose-dependent manner. The WB assay indicated a similar result to qRT-PCR; TRPM_2.5_ induced a marked increase in the protein levels of LC3II/LC3I and p62, and WIC was more significant than WSC (Figure 4D–F). These results suggest that TRPM_2.5_ and its components can activate autophagy in AC16 cells, with the effects of WIC being more pronounced than those of WSC.

### 2.4. PERK Gene Knockdown Alleviated Sensitivity of AC16 Cells to TRPM_2.5_

To characterize the potential involvement of the PERK signaling pathway in cell death caused by TRPM_2.5_, PERK was knocked down in TRPM_2.5_-treated AC16 cells using siRNA-PERK. The expression of *PERK* was measured by qRT-PCR and WB experiments after si-PERK transfection (Figure S1). The results clearly showed that si-PERK-1 exhibited better knockdown efficiency and was chosen for subsequent experiments.

**Figure 5:**
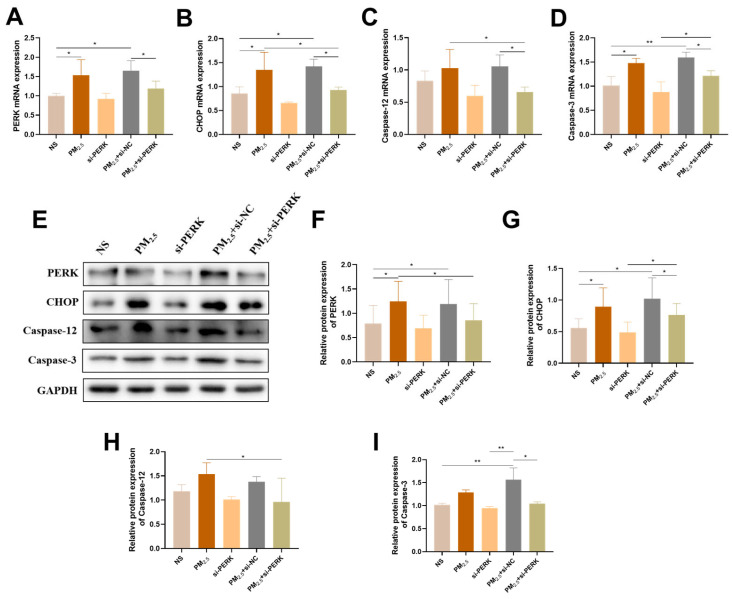
PERK knockdown alleviated TRPM_2.5_-induced ERS and apoptosis in AC16. (**A**–**D**) mRNA expression of PERK, CHOP, Caspase-12, and Caspase-3 in AC16. (**E**) Representative images of the relative protein expression of the above indicators. (**F**–**I**) Protein gray analysis of the above indicators. Data are shown as mean ± standard deviation (*n* = 3). * *p* < 0.05. ** *p* < 0.01.

Figure 5A–D shows that the mRNA levels of *PERK*, *CHOP*, *Caspase-12*, and *Caspase-3* were increased in the PM_2.5_ group, and such variations were strikingly suppressed in the PM_2.5_+si-PERK group. Similar phenomena were observed at the protein expression level (Figure 5E–I), which indicated that the PERK signaling pathway caused by TRPM_2.5_ promoted apoptosis in TRPM_2.5_-treated AC16 cells.

### 2.5. PERK/Sestrin2 Signaling Pathway Participated in the Initiation of Autophagy

**Figure 6:**
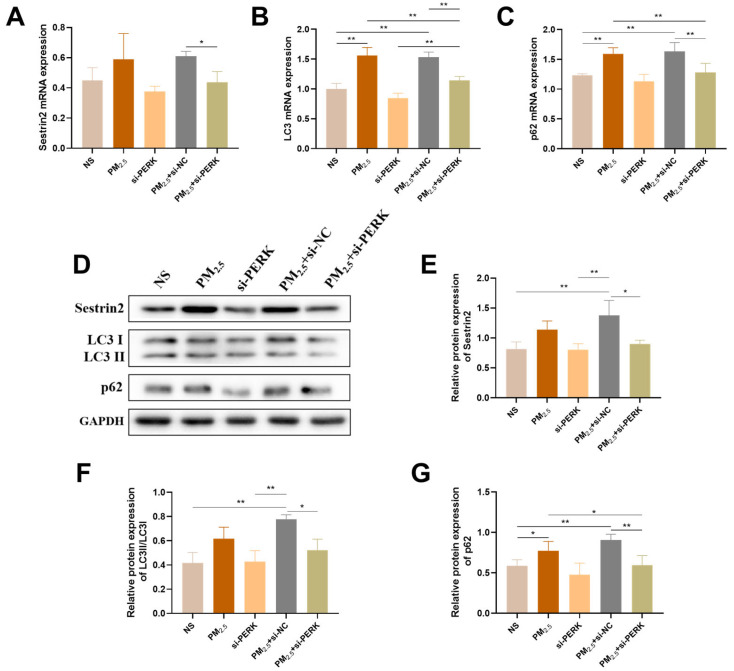
PERK knockdown mitigates Sestrin2 regulation of autophagy in TRPM_2.5_-treated AC16 cells. (**A**–**C**) mRNA expression of Sestrin2, LC3, and p62 in AC16 cells. (**D**) Representative images for the relative protein expression of the above indicators. (**E**–**G**) Protein gray analysis of the above indicators. Data are shown as mean ± standard deviation (*n* = 3). * *p* < 0.05. ** *p* < 0.01.

Sestrin2 is a novel stress-induced protein and positive regulator of autophagy. To further evaluate whether the endoplasmic reticulum stress PERK signaling pathway plays a crucial role in Sestrin2 regulation of autophagy, PERK gene silencing technology was utilized. The data showed that the mRNA enhanced effects of *Sestrin2*, *LC3*, and *p62* on TRPM_2.5_ activation were markedly abolished by PERK knockdown (Figure 6A–C). As expected, the activation of p62 at the protein expression level was largely reversed by siRNA-mediated knockdown of PERK (Figure 6D–G). These results suggest that the increase in PERK is responsible for Sestrin2 regulation of autophagy in TRPM_2.5_-treated AC16 cells.

### 2.6. Activation of Protective Autophagy Resists TRPM_2.5_-Induced Cell Damage

**Figure 7:**
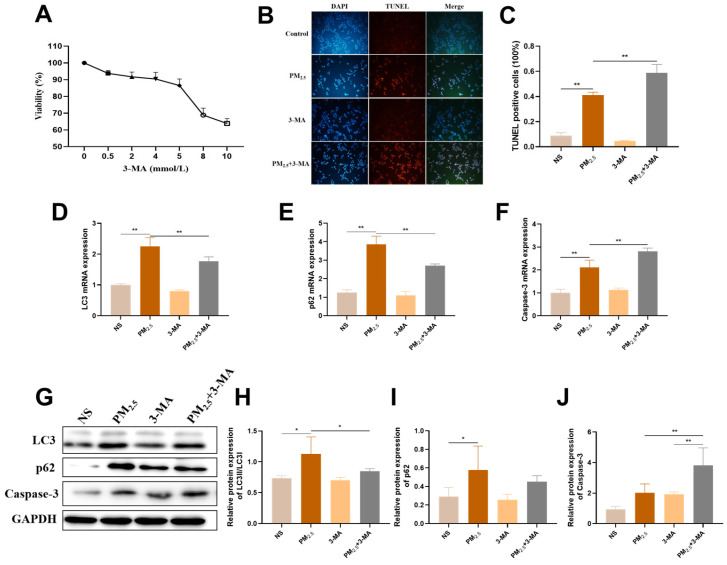
Protective autophagy was activated in response to TRPM_2.5_-induced apoptosis. (**A**) Cell viability of AC16 cells declined after 3-MA exposure. (**B**) TUNEL fluorescence staining map (×200). (**C**) TUNEL-positive cell rate in AC16 cells. (**D**–**F**) mRNA levels of LC3, p62, and Caspase-3 in AC16 cells. (**G**) Representative images of the relative protein expression of the above indicators. (**H**–**J**) Protein gray analysis of the above indicators. Data are shown as mean ± standard deviation (*n* = 3). * *p* < 0.05. ** *p* < 0.01.

To investigate the underlying role of autophagy in cell damage, the distinctive autophagosome formation inhibitor 3-MA was used on AC16 cells. The concentration of 3-MA that maintained cell viability above 90% was 4 mmol/L; therefore, it was incorporated in the following experiments (Figure 7A). The TUNEL experiment results showed that the percentage of TUNEL-positive cells in the PM_2.5_ group was significantly higher than that in the control group. Notably, under the intervention of 3-MA, this phenomenon was evidently exacerbated, indicating that inhibition of autophagy has a negative effect on cell apoptosis induced by TRPM_2.5_ (Figure 7B,C). Furthermore, qRT-PCR results revealed that pre-treatment with 3-MA suppressed autophagy-associated markers (i.e., *LC3* and *p62*), while the apoptosis-associated marker (*Caspase-3*) was comparatively upregulated (Figure 7D−F). Consistently, we also found that it had the same regulatory effect at the protein expression level (Figure 7G–J). Therefore, these findings confirmed that autophagy exerts a positive effect on TRPM_2.5_-induced cytotoxicity, as shown in the 3-MA pre-treatment group treated with TRPM_2.5_.

## 3. Discussion

Adequate evidence indicates that PM_2.5_ can cause heart damage; however, the potential mechanisms by which PM_2.5_ and its components induce cardiotoxicity remain to be fully elucidated. In the present experiment, we extracted WSC and WIC from TRPM_2.5_ to explore the specific mechanisms by which TRPM_2.5_ and its components induce autophagy and apoptosis in cardiomyocytes via ERS. We also investigated the important role of autophagy in this process was extensively probed. These results may provide new insights into the prevention and control of cardiovascular diseases after exposure to PM_2.5_.

Firstly, based on our experimental results, we chose 50 and 100 μg/mL of TRPM_2.5_, WSC, and WIC for a 24 h treatment period. These concentrations were found to result in a cell viability of approximately 80%, which allowed us to observe significant biological responses while maintaining a reasonable level of cell survival for further investigations. In comparison with previous literature, Yang et al. selected doses of 25, 50, and 100 μg/mL of PM_2.5_ to study the cytotoxic effects on AC16 cells [29]. Their findings indicated that these doses were effective in revealing potential toxicological mechanisms without completely compromising cell viability. For WSC and WIC, Qi et al. [30] found in their study that exposure to 50 µg/mL of Total-PM_2.5_ or WIC could significantly reduce the viability of neonatal rat cardiomyocytes (NRCMs), induce damage to the cell membrane, and increase the level of ROS. However, cytotoxicity induced by exposure to the WSC was not observed until the concentration was increased to 75 µg/mL. Although there are limited direct studies on the effects of different components on AC16 cells at specific concentrations, the selection of 50 μg/mL and 100 μg/mL of TRPM_2.5_, WSC, and WIC for a 24 h treatment period in our study was based on both our experimental results, aiming for a cell viability of approximately 80%, and relevant literature, ensuring that the chosen doses were appropriate for exploring the effects of these substances on AC16 cells and had a reasonable scientific basis.

Our research findings showed that TRPM_2.5_ and its different components can induce ERS, autophagy, and apoptosis in cardiac myocytes. As TEM is a widely used standard method for observing subcellular structural morphology, the first step in this study was to conduct TEM observations of the cell samples. Apparently, significant ER swelling and fragmentation, as well as excessive formation of autophagosomes, were observed by TEM following TRPM_2.5_ and component exposure. This result, together with the levels of ERS- and autophagy-related genes and proteins (specifically, GRP78, PERK, CHOP, Caspase-12, LC3, and p62) detected in this study, confirms that TRPM_2.5_ and its components induce the occurrence of ERS and autophagy. Among these, the PERK signaling pathway plays an important role. In previous studies, numerous reports have shown that the activation of PERK leads to eIF2α phosphorylation, which is a critical step in modulating protein synthesis and initiating downstream signaling cascades. The selective increase in ATF4 translation following eIF2α phosphorylation is also a well-documented phenomenon. Although we lack direct evidence of eIF2α phosphorylation and ATF4 levels in our current dataset, previous studies have found that PM_2.5_ can induce activation of the PERK/eIF2α/ATF4/CHOP pathway in HaCaT Human Keratinocytes [18]. Additionally, other research has reported that PM_2.5_ exacerbates airway inflammation and apoptosis by activating the PERK/eIF2α/ATF4/CHOP pathway associated with ERS in chronic obstructive pulmonary disease (COPD) [31]. In addition, CHOP and Caspase-12 are implicated as common downstream signaling molecules in various ERS-mediated apoptotic signaling pathways, and together with the changes in Caspase-3 (the ultimate executor of apoptosis) expression levels detected in our experiment, which demonstrated that TRPM_2.5_ and its components induced apoptosis, may be partially attributed to ERS. Similarly, Wang et al. found that PM_2.5_ can induce the activation of CHOP, Caspase-12, pro-Caspase-3, and cleaved-Caspase-3 in endothelial cells [32]. To further validate the above conclusions, the ERS-related molecule PERK was knocked out in AC16 cells, and subsequently, apoptosis- and autophagy-related molecules were significantly reversed. These results prove that TRPM_2.5_ induced AC16 cell autophagy and apoptosis through ERS.

Our data showed that TRPM_2.5_, WSC, and WIC stimulated the activation of Sestrin2. Sestrin2, a hypoxia-inducible gene 95, is considered a pivotal element in maintaining cellular stress-related homeostasis [33]. A wealth of evidence has pointed to the fact that Sestrin2 can regulate autophagy through multiple pathways and is also promoted by multiple stress injuries. Considering the rare reports of Sestrin2 on PM_2.5_, the underlying mechanisms were further elucidated by silencing PERK in AC16 cells. Our research findings revealed that Sestrin2, activated by PM_2.5_, was significantly inhibited in the PM_2.5_+si-PERK group, indicating that TRPM_2.5_ regulates the expression of Sestrin2 through the PERK signaling pathway, leading to autophagy in AC16 cells. Sestrin2/Nrf2 signal activation reportedly acts as an immediate downstream factor of the PERK pathway in combating ERS, which can improve the maturation of porcine oocytes, in line with our findings [34]. Based on the above results, Sestrin2 plays an indelible role in TRPM_2.5_-induced autophagy through ERS. From a theoretical perspective, multiple signaling pathways are known to be intertwined with autophagy regulation. For example, the mTOR pathway is a key regulator of autophagy and can interact with the ERS response pathway, where PERK is a central component. Another potential pathway is the PI3K/Akt pathway. This pathway is involved in a wide range of cellular processes, including cell survival, growth, and metabolism. Activation of the PI3K/Akt pathway can inhibit autophagy in some cases. In addition, the AMPK pathway, which senses cellular energy status, is an important regulator of autophagy. AMPK activation promotes autophagy by phosphorylating and activating autophagy-related proteins. Although we did not experimentally explore these additional signaling pathways in the current work, our findings provide a foundation for future investigations.

The current literature reveals that autophagy is significantly triggered by TRPM_2.5_ and its components. However, autophagy is a double-edged sword, making it challenging to discern its actual role in this context. It has been reported that autophagy has the potential to facilitate cell death via excessive self-digestion and degradation of essential cellular components [35]. While other studies have reported that autophagy is induced for cell survival. In order to determine the exact impact of autophagy in our study, 3-MA, an inhibitor of autophagy, was utilized to observe apoptosis. After pre-treatment with 3-MA, cell apoptosis became more severe to some extent, indicating that protective autophagy was activated by the TRPM_2.5_-induced PERK/Sestrin2 signaling pathway. Similar to previous studies, autophagy played a protective role against PM_2.5_-triggered inflammation in COPD by evoking macrophage autophagy [27]. Therefore, we concluded that autophagy was activated for cell survival in this study. In our study, the evaluation of apoptosis primarily relied on methods such as caspase-3 activation assays and TUNEL staining. Caspase-3 activation assays detect late events in apoptosis, potentially missing the early stages. Although TUNEL staining identifies DNA fragmentation associated with apoptosis, it may also have some degree of non-specificity. Given the limitations of our current apoptosis assessment methods, Annexin V detection should be incorporated in future studies to improve the accuracy of apoptosis assessment.

Our research findings indicated that the full particles of TRPM_2.5_ exhibit the strongest toxic effect on AC16 cells, followed by WIC, and WSC exhibited the weakest toxic effect. It is noteworthy that the cardiotoxicities of TRPM_2.5_, WSC, and WIC were estimated in AC16 cells. The results indicated that when the concentration reached 100 µg/mL, exposure to TRPM_2.5_ and WIC reduced the cell survival rates to 70–80%. However, cell viability remained at 84.7% until WSC was elevated to a higher concentration (400 µg/mL). In addition, WIC exhibited stronger cytotoxicity than WSC in terms of ERS, autophagy, and apoptosis. Our data indicated that WSC had lower cytotoxicity toward AC16 compared to TRPM_2.5_ and WIC, which is in line with the results reported by others in Beijing [36]. A study conducted in Taiyuan, China, found that water-soluble inorganic ion is the predominant component of water-soluble components of PM_2.5_ (WS-PM_2.5_), and the concentrations of heavy metals and PAHs are much higher in water-insoluble components of PM_2.5_ (WIS-PM_2.5_). They also found that WIS-PM_2.5_ is more toxic than WS-PM_2.5_ [30]. In our experiments, TRPM_2.5_ was also collected from Taiyuan, China, and the organic elements and water-soluble ions were extracted, and the amounts were analyzed. We analyzed and found that the mass of organic elements and water-soluble ions in the total TRPM_2.5_ mass accounted for 17.91% and 59.50%, respectively [4]. A study in Nanjing, China [37] found that the toxicity of substances from transportation sources to cells was higher than that of substances from industrial and residential sources. Among them, water-soluble ions accounted for 75.78% of PM_2.5_ from transportation sources, making them the largest contributor to the PM_2.5_ mass concentration. In addition, OC and EC accounted for a significant proportion of PM_2.5_, ranging from 7.35% to 15.30%, which was similar to our data, proving that our study has a certain representativeness.

However, we need to recognize several limitations of this study. First, the composition of PM_2.5_ varies significantly among different geographical locations, seasons and emission sources. The PM_2.5_ samples used in this study may not fully represent the wide variety of PM_2.5_ in the environment. This variability in composition may have an impact on the observed effects and underlying mechanisms. Second, considering the complex interactions among multiple organ systems in the in vivo environment, the PERK/Sestrin2 signaling pathway and protective autophagy triggered by TRPM_2.5_, WSC, and WIC are likely to behave differently. The findings of our study cannot be directly applied to the physiological environment in vivo. Therefore, future research should first conduct in vivo intervention experiments to confirm the current research findings. Despite these limitations, this study provides an important mechanism for evaluating the cardiotoxicity of TRPM_2.5_ and its components.

## 4. Materials and Methods

### 4.1. TRPM_2.5_ Collection and Extraction

During the period from November 2020 to January 2021, a high-flow sampler (Tianhong, Wuhan, China) equipped with a fiberglass filter membrane was used at a traffic intersection in Taiyuan City, Shanxi Province. The sampler was placed at the height of the breathing zone, and TRPM_2.5_ was collected from 8:00 to 20:00 at a flow rate of 1 m^3^/min. Sampling was paused during extreme conditions, such as snow, rain, and strong winds. The method for extracting TRPM_2.5_ was performed as described in our previous research [38].

In the early stage, we found that the average concentration of 16 polycyclic aromatic hydrocarbons in the collected TRPM_2.5_ was 159.67 ng/m^3^, accounting for 2.74% of the total mass of TRPM_2.5_. The average concentration of the five soluble anions was 28.22 μg/m^3^, accounting for 20.25% of the total mass of TRPM_2.5_ [4].

### 4.2. Cell Culture and TRPM_2.5_ Exposure

Human cardiomyocytes (AC16) were provided by Professor Junchao Duan, Capital Medical University. Cardiomyocytes were cultured in DMEM/F-12 medium (Seven, Beijing, China) containing 10% fetal bovine serum (FBS) (VivaCell, Shanghai, China), 1% penicillin-streptomycin (Seven, Beijing, China), and kept in an incubator containing 5% CO_2_ at 37 °C.

The weighted particles were suspended in PBS solution at a concentration of 5 mg/mL. Half of the suspension was used for the purpose of total-TRPM_2.5_. The remaining half of the solution was centrifuged at 3000 rpm for 20 min. The supernatant was then retrieved and employed as the WSC solution. To prepare the WIC solution, an amount of PBS equal in volume to the WSC was added to the precipitate. Subsequently, a certain volume of solution was removed and added to the culture medium to prepare 50 μg/mL PM_2.5_, 100 μg/mL PM_2.5_, 50 μg/mL WSC, 100 μg/mL WSC, 50 μg/mL WIC, and 100 μg/mL WIC.

AC16 cells were seeded in culture containers at a density of 1 × 10^5^ cells/mL. After the cells adhered, they were exposed to the prepared PM_2.5_ and its different component solutions for 24 h. PM_2.5_ was sonicated for 30 min before cell exposure. In the step treated with 3-MA, cells were pretreated with 4 mmol/L 3-MA (MedChemExpress, Monmouth Junction, NJ, USA) for 1 h before exposure to PM_2.5_.

### 4.3. Assessment of Cell Viability

The cytotoxicities of PM_2.5_, WSC, and WIC were evaluated using the Cell Counting Kit-8 (APExBIO, Houston, TX, USA). In brief, AC16 cells were incubated with 0, 12.5, 25, 50, 100, 200, and 400 μg/mL of PM_2.5_, WSC, and WIC for 24 and 48 h in 96-well culture plates. To confirm the effect of 3-MA on cell viability, AC16 cells were incubated with 0, 0.5, 2, 4, 5, 8, and 10 mmol/L of 3-MA for 24 h in 96-well culture plates. Subsequently, 10 μL of the CCK-8 solution was dispensed into each well and incubated at 37 °C for an additional 2 h. Finally, the absorbance was measured using a microplate reader at 450 nm.

### 4.4. Ultrastructure Observation

AC16 cells were plated in 6-well culture plates and treated with PM_2.5_, WSC, and WIC for 24 h to observe morphological and ultrastructure changes. Thereafter, the cells were washed with PBS and fixed with 2.5% glutaraldehyde for 5 min in the dark at room temperature. The cells were scraped off gently with a cell scraper, harvested, and fixed for 2 h at room temperature with 1% osmic acid in 0.1 M PBS. AC16 samples were subjected to dehydration using a graded ethanol series (30%, 50%, 70%, 80%, 95%, 100%, and 100%) and 100% acetone twice, embedded in 812 embedding agent, and sliced in an ultramicrotome. Ultrathin sections were stained with a 2% uranyl acetate-saturated alcohol solution and 2.6% lead citrate, and then observed under a TEM.

### 4.5. Immunofluorescence (If)

AC16 cells were plated onto 12-well chamber slides and exposed to PM_2.5_, WSC, and WIC for 24 h to determine the expression of GRP78. After processes, cells were cleaned using PBS, and subsequently, the cells were fixed with 4% paraformaldehyde for 20 min. Following this, the cells were permeabilized using 0.1% Triton X-100 for 20 min. Additionally, the cells were blocked with goat serum for 60 min at 37 °C, after which they were treated overnight at 4 °C with GRP78 antibody (1:1000, Proteintech Group, Wuhan, China). Samples without the primary antibody were used as negative controls to rule out interference from non-specific fluorescence. Then, the slides were rinsed and incubated with the corresponding FITC-conjugated AffiniPure Goat Anti-mouse IgG (H + L) (1:100, Boster, Wuhan, China) at room temperature for 1 h in the dark. Anti-fluorescence quencher (containing DAPI) was dropped onto the glass slides, after which images were captured using a fluorescence microscope and analyzed using Image-Pro Plus 6.0 software.

### 4.6. Terminal Deoxynucleotidyl Transferase dUTP Nick End Labeling (TUNEL) Staining

Similarly, cells were plated onto 12-well chamber slides and exposed to PM_2.5_, WSC, and WIC for 24 h, and TMR (Red) TUNEL Cell Apoptosis Detection Kit (Servicebio, Wuhan, China) was used to measure the apoptosis. We meticulously followed the procedures according to the manuals supplied by the manufacturer. Subsequently, the specimens were observed under a fluorescence microscope, and images were acquired. Both apoptotic and non-apoptotic cells could be stained blue by DAPI, but only red fluorescence was localized by TMR-5-dUTP incorporation in the apoptotic cell nucleus. Three images were randomly selected 3 images from the cell specimens. The TUNEL-positive cell rate was calculated as the number of apoptotic-positive cells divided by the total number of cells.

### 4.7. Cell Transfection

AC16 cells were cultured in 6-well plates, and low expression of PERK (si-PERK) and si-NC were transfected into AC16 cells when the cell density reached 70–80%. All processes were carried out according to the instructions of Lipofectamine 2000 (Invitrogen, Carlsbad, CA, USA). Briefly, Lipofectamine 2000 and siRNA were diluted using Opti-MEM medium, and the diluted siRNA and Lipofectamine 2000 were blended and incubated at room temperature for 5 min. The mixture was then added to the cell culture. Transfection was carried out for 6 h, after which the medium was changed to a complete medium, and the cells were cultivated for 12 h before PM_2.5_ exposure. The primer sequences of siRNA are listed in Table S2.

### 4.8. Quantitative Real-Time PCR (qRT-PCR) Analysis

RNAiso Plus was used to extract total RNA from AC16 cells (TaKaRa, Kusatsu, Japan), and the synthesis of cDNA was completed using PrimeScript^TM^ RT reagent kits (TaKaRa, Kusatsu, Japan). qRT-PCR was performed using TB Green Premix Ex Taq^TM^ II (TaKaRa, Kusatsu, Japan) on an Applied BIO-RAD CFX Connect Real-Time System (Hercules, CA, USA) by way of an initial denaturation at 95 °C for 30 s, followed by 40 cycles of amplification, each cycle consisting of 95 °C for 5 s and 60 °C for 30 s, then 95 °C for 15 s, 60 °C for 30 s and 95 °C for 15 s. The primer sequences of the detected genes are listed in Table S1. The relative quantities of genes were normalized to β-actin, and the 2^−ΔΔCt^ method was used for data processing.

### 4.9. Western Blotting (WB) Analysis

To evaluate total protein expression, lysates of AC16 cells were prepared, and the protein concentrations were determined using an BCA protein assay kit (Boster, Wuhan, China). Four times the amount of total lysate was mixed with 5×SDS-PAGE protein loading buffer. The samples were then boiled for 10 min. Subsequently, SDS-PAGE was utilized to separate the components, which were then transferred onto PVDF membranes. In a TBST solution containing 5% skimmed milk, the membranes were blocked for 2 h and incubated with primary antibody dilution in freshly prepared PBST with 1% skimmed milk overnight with gentle agitation at 4 °C. The primary antibodies used were GRP78 (1:10,000), PERK (1:1000), and LC3 (1:2000) (both purchased from Proteintech Group, Wuhan, China), p62 (1:1000, CST, Danvers, MA, USA), CHOP (1:1000, HuaAn Biotechnology, Hangzhou, China), Caspase-3 (1:2000), Sestrin2 (1:1000) (both purchased from Abcam, Cambridge, UK), Caspase-12 (1:800), β-actin (1:4000) (both purchased from Signalway Antibody, College Park, MD, USA), and GAPDH (1:5000, ABclonal Technology, Wuhan, China). Samples without added primary antibodies were used as negative controls for detecting non-specific binding background signals. After washing, the membranes were incubated at room temperature for 40 min with an anti-mouse/rabbit secondary antibody (1:8000, Boster, China). Protein bands were displayed using a chemiluminescence reagent (Boster, China), and the images were scanned and analyzed using ImageJ 1.8.0 software. Protein loading was controlled by probing with GAPDH/β-actin protein.

### 4.10. Statistical Analysis

Statistical analysis was performed using SPSS 22.0. Data are presented as mean ± standard deviation, and one-way ANOVA, followed by the LSD post-hoc test, was employed to examine the differences among groups. Probability levels of * *p* < 0.05 and ** *p* < 0.01 were considered statistically significant.

## 5. Conclusions

In conclusion, our study provides robust scientific evidence that exposure to TRPM_2.5_, WSC, and WIC can lead to heart damage, with autophagy induced by the PERK/Sestrin2 pathway playing a crucial role in the stimulation induced by TRPM_2.5_ and its components. Our research opens new possibilities for developing targeted therapeutic interventions. Furthermore, our component analysis results suggest that WIC-TRPM_2.5_ exposure may have more detrimental effects on the heart than WSC-TRPM_2.5_ through multiple mechanisms. This insight is valuable for environmental risk assessment and policymaking. Overall, our findings not only offer a novel perspective on the mechanisms and active ingredients of TRPM_2.5_ in heart disease but also have practical implications for preventing cardiac damage caused by environmental toxins. This paves the way for future research aimed at translating these findings into clinical and public health applications to safeguard cardiovascular health.

## Data Availability

Data is contained within the article and Supplementary Materials.

## Supplementary Materials

The following supporting information can be downloaded at: https://www.mdpi.com/article/10.3390/ijms26083784/s1.
