# Supplementary material for: PERK/Sestrin2 Signaling Pathway Mediated Autophagy Regulates Human Cardiomyocytes Apoptosis Induced by Traffic-Related PM2.5 and Diverse Constituents
Source: Int J Mol Sci. 2025 Apr 17;26(8):3784. doi: 10.3390/ijms26083784 (PMC12027522; doi:10.3390/ijms26083784)
Supplement: Supplementary file 1 [file ijms-26-03784-s001.zip › ijms-3551298-supplementary.pdf]

Table S1 The primer sequences used for qRT-PCR

Gene	Primers	Species	Sequences (5'-3')
GRP78	Forward	Human	CATGGTTCTCACTAAAATGAAAGG
GRP78	Reverse	Human	GCTGGTACAGTAACAACTG
PERK	Forward	Human	AGATTCTGGCTGTGATAATGCTTCC
PERK	Reverse	Human	TTTGCTACTGGTGGGCTTGAAAG
CHOP	Forward	Human	GCCTTTCTCCTTTGGGACACTGTCCAGC
CHOP	Reverse	Human	CTCGGCGAGTCGCCTCTACTTCCC
Caspase-12	Forward	Human	AACAACCGTAACTGCCAGAGT
Caspase-12	Reverse	Human	CTGCACCGGCTTTTCCACT
Caspase-3	Forward	Human	GACAGACAGTGGTGTTGATGATGAC
Caspase-3	Reverse	Human	GGCACAAAGCGACTGGATGAAC
LC3	Forward	Human	TACGGAAAGCAGCAGTGT
LC3	Reverse	Human	GAAGGCAGAAGGGAGTGT
p62	Forward	Human	GCACCCCAATGTGATCTGC
p62	Reverse	Human	CGCTACACAAGTCGTAGTCTGG
Sestrin2	Forward	Human	GGCTCATCACCAAGGAACACATC
Sestrin2	Reverse	Human	AGCCAAACACGAAGGAGGAGAG
β-actin	Forward	Human	CCTGGCACCCAGCACAAT
β-actin	Reverse	Human	GGGCCGGACTCGTCATAC

Table S2 The primer sequences of si-PERK

Gene	Sequences	
	Sense (5'-3')	Antisense (5'-3')
si-PERK-1	GCCACUUUGAACUUCGGUAUATT	UAUACCGAAGUUCAAAGUGGCTT
si-PERK-2	GCUUUGGAAUCUGUCACUAAUTT	AUUAGUGACAGAUUCCAAAGCTT
si-PERK-3	CCUCAAGCCAUCCAACAUAUUTT	AAUAUGUUGGAUGGCUUGAGGTT

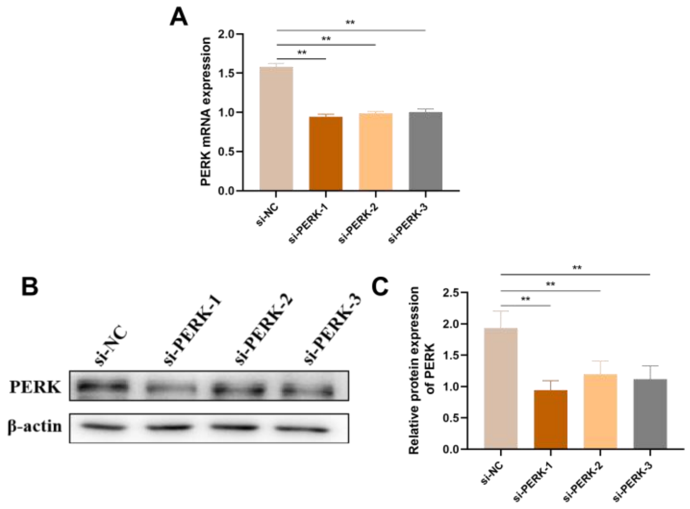

Fig. S1. The PERK gene silencing of identification in AC16 cells. The proliferating AC16 cells were transfected with si-PERK (S1, S2, S3) or si-NC using Lipofectamine 2000. (A) The mRNA expression of PERK in AC16 cells. (B) Representative images of western blotting for the relative protein expression of PERK. (C) The protein gray analysis of PERK. Data are expressed as the means ± SD (n = 3). $^{*}P < 0.05$. $^{**}P < 0.01$.
